# Strengthening surveillance in Ghana against public health emergencies of international concern

**DOI:** 10.1186/s41182-022-00473-w

**Published:** 2022-10-28

**Authors:** Motoi Adachi, Kiyosu Taniguchi, Hiroki Hori, Taketoshi Mizutani, Aya Ishizaka, Koichi Ishikawa, Tetsuro Matano, David Opare, Doris Arhin, Franklin Bekoe Asiedu, William Kwabena Ampofo, Dorothy Manu Yeboah, Kwadwo Ansah Koram, Abraham Kwabena Anang, Hiroshi Kiyono

**Affiliations:** 1 St Mary’s Hospital, 422 Tsubukuhonmachi, Kurume, Fukuoka 830-8543 Japan; 2 National Mie Hospital, Tsu, Japan; 3 Mie University, Tsu, Japan; 4 Graduate School of Frontier Science, The University of Tokyo, Tokyo, Japan; 5 The Institute of Medical Science, The University of Tokyo, Tokyo, Japan; 6 AIDS Research Centre, National Institute of Infectious Diseases, Tokyo, Japan; 7 Joint Research Center for Human Retrovirus Infection, Kumamoto University, Kumamoto, Japan; 8 Ghana Health Service, Accra, Ghana; 9 Noguchi Memorial Institute for Medical Research, University of Ghana, Accra, Ghana; 10 Institute for Global Prominent Research, Graduate School of Medicine, Chiba University, Chiba, Japan; 11 CU-UCSD Center for Mucosal Immunology, Allergy and Vaccines (cMAV), Department of Medicine, University of California San Diego, San Diego, CA USA

**Keywords:** Surveillance, Public health emergencies of international concern (PHEIC), Diarrhea pathogens

## Abstract

Among western African countries, the Republic of Ghana has maintained an economic growth rate of 5% since the 1980s and is now categorized as a middle-income country. However, as with other developing countries, Ghana still has challenges in the effective implementation of surveillance for infectious diseases. Facing public health emergencies of international concern (PHEIC), it is crucial to establish a reliable sample transportation system to the referral laboratory. Previously, surveillance capacity in Ghana was limited based on Integrated Disease Surveillance and Response, and therefore the “Surveillance and Laboratory Support for Emerging Pathogens of Public Health Importance in Ghana (SLEP)” was introduced to strengthen diarrhea surveillance. The SLEP project started with a sentinel diarrhea survey supported by SATREPS/JICA in collaboration with National Public Health Reference Laboratory (NHPRL) and Noguchi Memorial Institute of Medicine (NMIMR). The base-line survey revealed the limited capacity to detect diarrhea pathogens and to transfer samples from health centers to NHPRL. The involvement of private clinic/hospital facilities into the surveillance network is also crucial to strengthen surveillance in Ghana. The strong and interactive relationship between the two top referral laboratories, NHPRL under the Ministry of Health NMIMR and under the Ministry of Education, enables Ghana Health Services and is critical for the rapid response against PHEIC. In future, we hope that the outcome of the SLEP surveillance project could contribute to building a surveillance network with more timely investigation and transfer of samples to referral labs.

## Background

**Fig. 1:**
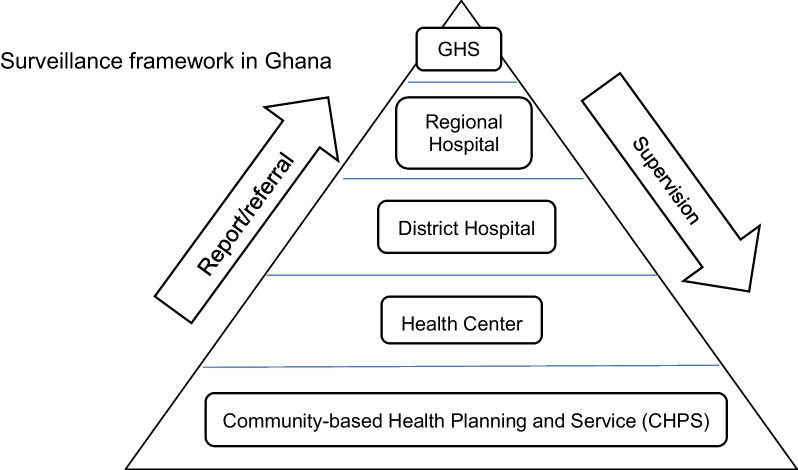
Surveillance structure in Ghana. Surveillance framework in Ghana. The surveillance system is divided into several levels. Integrated Disease Surveillance and Response (IDSR) shapes the structure. Community-based Health Planning and Services (CHPS), which is the most grass-roots level, starts the reporting of priority diseases towards Ghana Health Services, the apex facility in Ghana’s IDSR system. Feedback (supervision and training) is provided by higher-level facilities to facilities in the level below

The Republic of Ghana is a western African country, facing the Gulf of Guinea, and has maintained an economic growth rate of 5% since the 1980s. In 2010, an offshore oilfield was discovered and Gross National Income (GNI) reached $1,190 in 2019, which categorized it as a middle-income country [1]. In the health sector, rural community-based health planning and services (CHPS) have been adopted since the 1990s, which consisted of three layers: “Community”, “Health center” and “District Hospital”. Ghana Health Services (GHS) has planned and implemented CHPS since 2005 (CHPS Operational Policy) on a country-wide scale [2]. A CHPS facility is built for every 1500–4500 residents and these are the frontline of health care provision and disease surveillance in Ghana. When a priority disease such as cholera, dengue or measles starts to spread, clinics at the CHPS level or health centers report it to the next facility in the CHPS network, the district hospital [3]. Then, district hospitals report it to the regional hospital and regional hospitals report it to GHS. WHO Regional Office for Africa (AFRO) has made the best of a layered health system and established Integrated Disease Surveillance and Response (IDSR) to implement surveillance effectively [4] (Fig. 1).

**Fig. 2:**
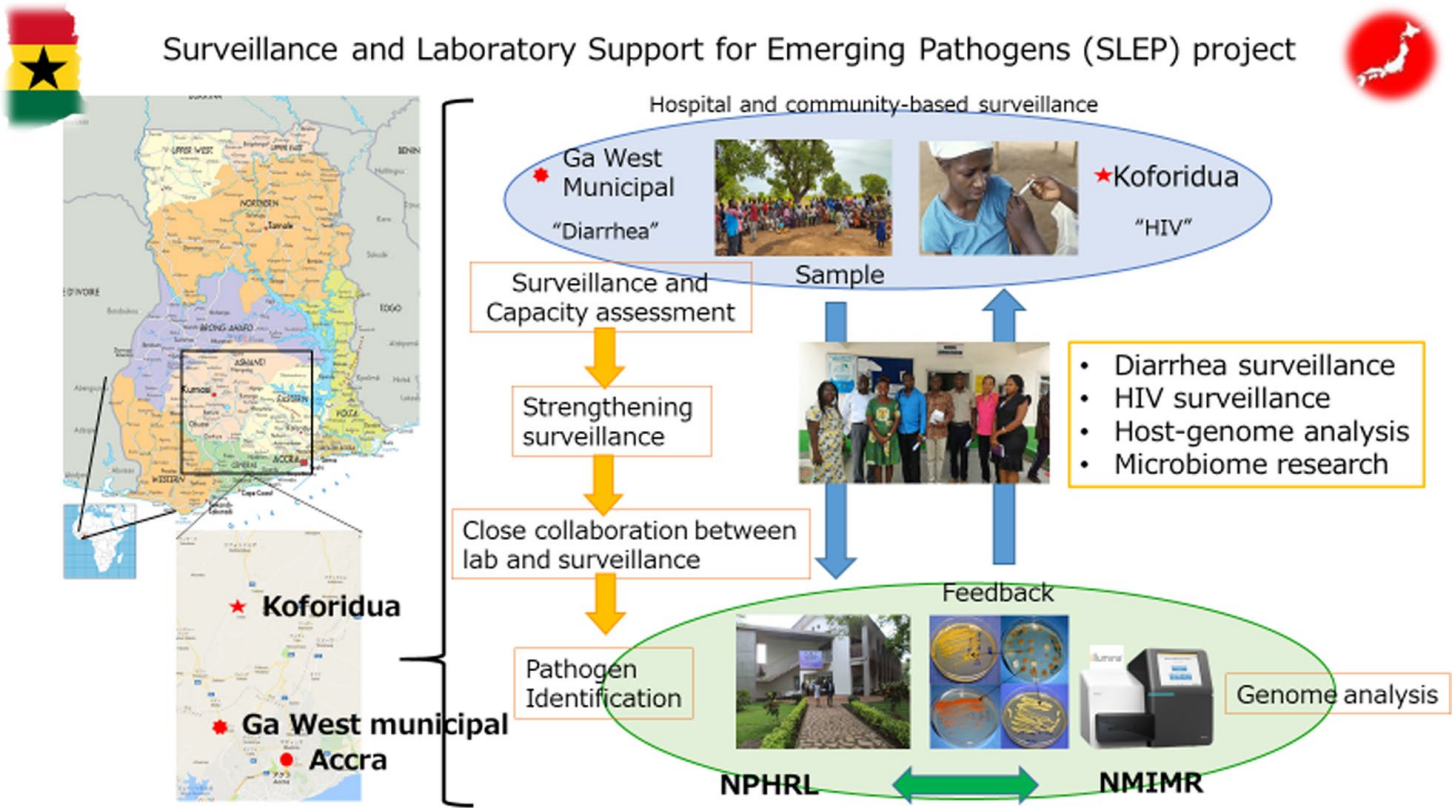
The overview of the SLEP project. There are two arms to this project. One is to strengthen surveillance directly through the diarrhea sentinel survey and the other is to identify whether certain microbiome profiles are more commonly observed in patients with severe diarrheal disease in Ghana

Since the Republic of Ghana is located in a tropical wet climate, tropical diseases such as malaria are endemic, and cholera outbreaks occurred in 2014 and 2016 [6]. In 2016, an Ebola hemorrhagic fever outbreak occurred in central Africa and WHO declared it to be a public health emergency of international concern (PHEIC). The Republic of Ghana needed to strengthen the existing surveillance system and requested assistance from Japan. Through the International Collaborative Research Program, Science and Technology Research Partnership for Sustainable Development (SATREPS) supported by Japan International Cooperation Agency (JICA)/Japan Agency for Medical Research and Development, the SLEP project (Fig. 2) was established in 2016. In this project, a fixed-point (sentinel) survey was newly introduced to investigate the intestinal pathogens of diarrhea diseases, with the strong support of GHS. Before implementing the diarrhea survey, the existing surveillance capacity was evaluated as the base-line survey.

### Activities

The diarrheal surveillance component of the SLEP project targeted Ga West Municipality, which is adjacent to the capital Accra and consists of a mix of densely populated areas where urban residents live along the main road and rural villages with no roads. Taking the geographical characteristics into account, the surveyed medical facilities were selected by balancing densely populated and non-densely populated areas. In addition to evaluating the implementation of IDSR as an indicator of surveillance, the Medical Facility Capacity Assessment Guidelines (partially revised according to the situation in Ghana) prepared by the WHO East Africa Office were employed [5]. Finally, 32 medical facilities were chosen (Table 1). The survey prepared questionnaires for district hospital level, health center level, CHPS level and clinical laboratories attached to these medical facilities. At least two surveyors visited the facility as a team and interviewed and checked the status visually, checking points such as the existence of IDSR manuals. In addition, 11 clinical laboratories (out of the 32 health facilities) were investigated to determine their ability to perform diarrheal disease testing and capacity to transfer specimens to referral labs. The laboratories were at the district hospital, two health centers, six private hospitals and two private clinics (Table 2). Regarding the diarrhea surveillance capacity, the surveyor visually confirmed the presence of stool test kits, and asked about the sample transport method.

**Table 1:** Availability of IDSR manuals in the 32 medical facilities participating in the SLEP project

Facility	IDSR manual identified	Public	Private	The total number of health facilities
District Hospital	1	1/1 100%	–	1
Health center	3	3/4 75%	–	4
Hospital	0	0 0%	0/6 0%	6
Polyclinic	0	0 0%	0/2 0%	2
Clinic	1	0/3 0%	1/7 14.3%	10
Maternity home	0	0 0%	0/4 0%	4
CHPS	1	1/5 20%	–	5
Total	6	5/13 38.5%	1/19 5.2%	32

Facilities are broken down by their level in the IDSR system, and whether they are public or private facilities

**Table 2:** Capacity of laboratories to perform diarrheal disease testing. The number and proportion of laboratories which have malaria and/or cholera rapid tests, dysentery exam kits, and the ability to transfer samples to the NPHRL are reported

	District hospital	Health center	Private hospital	Private clinic	Total
Malaria rapid test	1/1 100%	2/2 100%	6/6 100%	1/2 50%	10/11 90.9%
Cholera rapid test	0/1 0%	1/2 but expired, so 0%	3/6 50%	0/2 0%	4/11 36.4%
Dysentery exam (direct iodine method)	1/1 100%	2/2 100%	6/6 100%	1/2 50%	10/11 90.9%
Capacity to transfer samples to NPHRL	1/1 100%	0/2 0%	2/6 33.3%	0/2 0%	3/11 27.2%

## Results

The IDSR manual was used at only 5 out of 13 public and 1 out of 19 private health facilities (Table 1). Of the 11 laboratories investigated in the survey, including the district hospital, health centers, private hospitals and clinics, all 11 had rapid test kits for malaria diagnosis. Ten facilities had evidence of stool tests, while the number who transported specimens to the National Public Health Reference Laboratory, NPHRL was 3 out of 11 (27.3%). Cholera rapid test kits were provided in 4 out of 11 laboratories (36.3%). However, the cholera rapid kit had expired in the district hospital (Table 2).

Health facilities in the IDSR are expected to submit weekly and monthly surveillance reports. However, no weekly reports were submitted from private facilities. Also, only one of the private facilities received training from upper-level facilities, compared to 9 out of 13 of the public facilities (Table 3).

**Table 3:** Summary of facilities submitting weekly and monthly surveillance reports by the submission deadline, and the number of facilities which received training from upper-level facilities

	IDSR guideline	Weekly report	Monthly report	Training from upper-level facilities
Public facilities	5/13 38.4%	9/13 69.2%	10/13 76.9%	9/13 69.2%
Private facilities	1/19 5.3%	0/19 0%	9/19 47.3%	1/19 5.3%
Total	6/32 18.8%	9/32 28.1%	19/32 59.3%	10/32 31.3%

## Discussion

In Ghana, public health centers and clinics were systematically established based on the IDSR framework. However, in addition to logistical difficulties due to remoteness of areas, the surveillance still has challenges in efficient implementation and inter-facility cooperation [5, 6]. While almost all of the laboratories surveyed had access to diarrhea test kits (Table 2), the ability of laboratories to transport specimens to higher-level referral facilities was clearly a challenge.

Before beginning the SLEP project, public transportation, mailing, and police requests for transporting pathogen samples were tried in various regions in Ghana, with no improvement observed in the timely delivery of specimens [7, 8]. In this project, it was decided that public transportation should no longer be used, and that the project vehicle (four-wheel drive) should transfer samples to NPHRL and NMIMR from Ga West municipal.

A specimen transportation system was built to transport diarrheal samples investigated at NPHRL from Ga West municipal in Accra. Samples were split and sent to both NPHRL and NMIMR, with NPHRL performing campylobacter and salmonella species investigation in addition to cholera and dysentery and NMIRM performing the four bacterial cultures and PCR testing for rotaviruses and noroviruses, and metagenomic/microbiome analysis.

We also aimed to quickly and periodically provide feedback of the stool test results conducted at the top two referral facilities to Ga West district hospitals to aid in clinical care. Timely and reliable feedback on diarrheal testing results will strengthen surveillance from the clinical side and encourage cooperation of hospitals involved in the surveillance network. Finally, we distributed a revised IDSR manual to all health facilitates in Ga West to improve implementation of IDSR guidelines.

Since the IDSR guidelines were identified in 6 out of 32 facilities (18.8%), the project distributed the revised IDSR manual to all health facilities in Ga West.

Private health care facilities had fundamentally different attitudes from public ones towards surveillance. Private institutions are legally required to report surveillance records. However, because tax is imposed on the number of outpatients, private health facilities are said not to report all patients, which resulted in incomplete surveillance. Because the number of private health facilities is much bigger than that of public, certain measures to make private health facilities get involved in the surveillance should be taken in the administrative process. Training for health facilities at the grass-roots level from district hospitals would encourage the participation of private health facilities.

The smooth and reliable coordination between the top two referral laboratories was crucial. In the surveillance scheme in Ghana, investigations for PHEIC such as Ebola virus are conducted at NMIMR. On the other hand, cholera, HIV, and tuberculosis, which are categorized as priority diseases in IDSR, are inspected at NPHRL under the jurisdiction of the Ministry of Health. Therefore, the NPHRL has a significant role in the surveillance. NMIMR at the University of Ghana has been a key partner for JICA medical cooperation projects for many years, and is recognized as a top referral facility in West Africa. Since the NPHRL performs testing for the pathogens indicated as priority by the IDSR, NMIRM worked together with NPHRL on the SLEP project and performed quality control (double checking) as needed. Although previously cooperation between the NMIMR of Ghana University and the NPHRL was sparse, we are working to strengthen cooperation between the bacteriology department of NMIMR and NPHRL by collecting and storing fecal samples together. It is expected that this close cooperation will overcome administrative jurisdictions and organizational or cultural barriers. In the future, it is hoped that the divisional roles between the two organizations will be appropriately carried out.

In addition, NMIMR had a next-generation sequencer used in gut microbiota research that was used in this project, indicating the possibility of using research-based test data to enhance surveillance [9]. This project aims to strengthen infectious disease surveillance in Ghana by allowing the NPHRL to become independent and function as a national reference lab in the future. In order to achieve this goal, we are working to develop a cooperative system and develop human resources within Ghana's domestic institutions.

While the SLEP study focused on diarrheal disease, public health emergencies stipulated by IHR2005 include chemical, radiation, and food safety emergencies in addition to infectious diseases. Greater focus on these areas would be critical to strengthen Ghana’s ability to respond to PHEIC, as well as better collaboration with veterinary departments for surveillance against zoonotic diseases.

## Conclusions

In the face of PHEIC, it is critical to detect unusual events through surveillance and rapidly refer patient specimens to referral laboratories for confirmation. Surveys completed as part of the SLEP project identified key areas for enhancing the surveillance network in Ghana, such as streamlining the specimen transport system. A strong and interactive relationship between the two referral laboratories, NPHRL under the Ministry of Health and NMIMR under the Ministry of Education, will enable GHS to be more effective. In addition, greater involvement of private clinics and hospitals in the surveillance network should be prioritized to improve surveillance in Ghana.

## Data Availability

All tables are supported by the Report on baseline survey on Integrated Disease Surveillance and Response (IDSR) in Ga West municipality. Disease Surveillance Department, GHS in collaboration with JICA and Noguchi Memorial Institute of Medical Research 8.

## Abbreviations

GNI: Gross National Income
PHEIC: Public Health Emergencies of International Concerns
CHPS: Community-based health planning and services
IDSR: Integrated Disease Surveillance and Response
GHS: Ghana Health Services
AFRO: World Health Organization Regional Office for Africa
SATREPS: Science and Technology Research Partnership for Sustainable Development
NPHRL: National Public Health Reference Laboratory
JICA: Japan International Cooperation Agency
SLEP: Surveillance and Laboratory Support for Emerging Pathogens of Public Health Importance in Ghana
NMIMR: Noguchi Memorial Institute of Medical Research
PCR: Polymerase chain reaction
